# Healthcare and Physical Education of Children and Youth in Prague 1869–1914

**DOI:** 10.3389/fspor.2020.581285

**Published:** 2020-12-10

**Authors:** Marek Waic, Dagmar Pavlů

**Affiliations:** ^1^Department of Kinanthropology and Humanities, Faculty of Physical Education and Sport, Charles University, Prague, Czechia; ^2^Department of Physiotherapy, Faculty of Physical Education and Sport, Charles University, Prague, Czechia

**Keywords:** 19th century, Cisleithania, children, health care, physical education, legislation

## Abstract

The article focuses on the healthcare and physical education of children and youth in Prague, the capital city of Czech lands, in the period after the Austro-Hungarian compromise of 1867. The legislative framework for children's physical development and healthcare consisted of laws passed by the Imperial Council which were in force throughout the entire region of Cisleithania. Its execution and implementation, however, were the responsibility of the Czech territorial assembly and Prague municipality. The study analyses the environment in which children grew up, the quality of their diet, and their medical care, particularly the activities of school doctors. Further, the text concentrates on the organization and the quality of school physical education. Prague serves as an example of an industrial centre of the Cisleithanian region whose industrial development caused rapid urbanization which limited the possibilities of physical development of children and youth. Until the end of the 19th century, the only possibility of organized exercises was school physical education, and its quality was greatly influenced by the modest spatial conditions of schools. Even at the better-equipped grammar schools, physical education was an optional subject until 1909 and was not taught at most of them at all. As part of the modernization of the empire, the Cisleithanian government supported physical education, also for military reasons. The same was done by the Prague municipality, where care for the physical development and health of children and youth did not become the subject of political disputes.

## Introduction

The following study focuses on the Prague municipality's attention to the health and physical development of children and youth in the context of dynamic industrialization and urbanization and other social changes caused by these historical processes. The study also pays attention to the development of legislative norms that were valid throughout the Cisleithanian region and which created the legislative framework within which the municipal authorities channeled their efforts. The December Constitution, adopted in 1867, divided the Habsburg Monarchy into two independent states. Except for foreign policy, the army, currency, and the customs union, most of the legislative and executive power in Hungary passed into the hands of the Hungarian nobility and the administration of the so-called Cisleithania, to which the Czech lands (Bohemia, Moravia, Silesia) also belonged, remained in the hands of the Emperor and the Viennese government. According to the constitution, however, the central power gave the territorial assemblies the power to create legislative norms that could supplement the laws passed by the Imperial Council. The period covered by this study is also characteristic of the efforts of the Imperial Council and the Viennese government to modernize the entire Cisleithanian region. The social legislation and the school system, which are both very important for the physical development of children and young people, were dictated by imperial laws. But the imperial legislation did not interfere much in these particular areas and the practical implementation of the letter of the imperial laws was the responsibility of the municipal authorities. In healthcare, here the imperial laws applied too, but the execution of these laws and financing of healthcare was the responsibility of the Czech territorial assembly and Prague municipality. The implementation of a specific form of health care and physical development of children and youth was, therefore, primarily in the hands of municipal authorities.

The period after the Austro-Hungarian compromise is of particular interest because a few years later, in 1869, a law on primary and middle schools was passed in the Cisleithanian region which introduced compulsory physical education for all children for 2 h per week. This was a turning point in the history of the controlled physical development of children. From the 1860s until the late 1880s, school physical education for children was the only chance to do physical exercise in an organized way. Sokol, the largest Czech gymnastics association, started training children and teenagers only in the late 1880s.

The city of Prague was chosen as an example for the study of the controlled physical development of children and youth for the following reasons: Prague achieved almost the same dynamics of industrialization and urbanization as Vienna, but ethnic conditions in Prague, although a Czech-German city, were clearer and more homogeneous. As part of the economic migration of the inhabitants of the Cisleithanian region, a large number of workers, craftsmen, and women who served in the households of upper and middle classes, migrated temporarily, but also permanently, to the metropolis of the Habsburg monarchy. Vienna thus became a multi-ethnic city in the second half of the 19th century. Its political development was also more complex, and at the end of the century, it led to Germanization, which excluded members of the Slavic nations (Gletler, 1972, p. 293–299).

The political development of Prague in the period under review was simple. In 1861, the Czechs won the municipal elections and controlled the City Hall until the First World War. Although the municipal support, both moral and financial, was directed to Czech associations, the Czech councilors did not attempt to “Czechize” the city. They could not have done it even if they wanted because the Czech and German national powers were equal within the territorial assembly thanks to the strong German population of the bordering regions of Czech lands. Also, the bureaucrats of the Prague governance, which was subject to the Vienna Ministry of the Interior, would not allow the Czechization of Prague. The Prague municipality accepted the division of social and economic life of Czechs and Germans in the 1860s (Cohen, 2000, p. 47–71). Furthermore, Czech councilors respected the approximate parity of Czech and German schools in the city, which were financed by the territorial assembly, but also by the Prague municipality, because the Germans paid the same taxes to the municipal treasury as the Czechs. Prague City Hall was ruled by representatives of two Czech national parties who called themselves Old Czech Party and Young Czech Party. The goal of their political efforts, i.e., the political and cultural autonomy of the Czechs within the Cisleithanian region, was the same. How to achieve such autonomy, this is where they differed in opinion. However, they reached a mutual consensus on the municipal level at the Prague City Hall. The development of the city, the support of Czech schools, and also Czech associations, particularly Sokol, were understood by the representatives of both parties as necessary steps to strengthen the position of the Czechs in the metropolis of the Czech lands. In the 1860s, the creation of self-governing bodies of the municipality and the internal hierarchy of the City Hall were completed. Self-governing bodies and municipal officials paid particular attention to good management of municipal finances. The increase in the population of Prague brought about by fast industrialization led to rapid urbanization, and from the 1870s, the city had to invest in its infrastructure—remediation, expansion of the street network, installation of the street lights, and construction of the water supply system so that the inhabitants had drinking water (Slámová, n.d.).

The Prague City Council cooperated with external experts, especially school physicians and school principals. Of course, the physical education of young people was not one of the priorities of the Prague City Hall, but the representatives were close to the Sokol organization. Two mayors (Tomáš Cerný—Mayor in 1882–1885 and Jan Podlipný—Mayor in 1897–1900) even headed the Sokol organization.

The health and physical development of children and young people living in Prague were significantly affected by their living conditions, diet, medical care, and physical exercise opportunities, especially at school, as well as their protection by labor legislation, and therefore this article also focuses on these factors. The comparison of children and youth welfare with other provincial metropolises would be very interesting and undoubtedly beneficial for the history of Cisleithania, but the prescribed scope of the study does not allow it.

## Methodology

In the period we analyzed, we also observed the interacting complex of phenomena (housing level, diet, labor legislation, development of the school system, the content of school physical education, child health care), which affected the physical activities of children and youth and their health. We have worked mainly with primary sources, especially with the texts of selected imperial laws, or their passages, which determined the organizational forms and content of health care and physical education of children and youth in the Cisleithanian region. A very important title of contemporary literature is the History of Prague Education 1860–1914. It is a comprehensive chronicle of Prague education in that period while also a great volume of contemporary resources. Its author, the director of public school and a contemporary witness, cites the legislative norms adopted by the territorial assembly and the decisions of the city council regarding Prague schools, including physical education, the activities of school doctors, etc. Another area of historical sources of the presented study became the texts of selected laws, or their passages, which formed the legislative framework for children and youth social and medical care. Other primary sources were the physical education curricula for primary and secondary schools and regulations for examinations of physical education teachers at secondary schools from the turn of the 19th and 20th centuries. The facts about the economic and social development in the Cisleithanian region and also about living conditions in Prague were taken from the most recent and extensive professional literature of Czech authors who deal with the history of Bohemia and its metropolis and to which we refer in the text.

## Demographic, Urban, and Social Changes in Prague, Affecting the Health and Physical Development of Children and Youth

The Czech lands were mostly Czech-German territory in terms of ethnic composition. The revolutionary demographic, social, and lifestyle changes that influenced and limited the development of physical education of children and youth took place in the Czech and German environment almost identically. However, the cultural emancipation of the Czech nation was very dynamic since the 1860s, as evidenced for example by the fact that around 94% of the Czech population of the Czech lands was alphabetized (Hlavačka, 2016, p. 199), which was crucial for the dissemination of physical and health education.

Industrialization, which achieved considerable development in the late 1860s in the Czech lands, brought significant changes in the social structure of society. This process is described by some authors as “decorporation,” i.e., the transition from the Estates Society to the Class Society (Jindra and Jakubec, 2015, p. 134), which is characterized by a society's division into the bourgeoisie and the working class and by the emergence of the middle class.

The conditions of life of the urban population and its lifestyle corresponded to the development of social stratification of the urban population through two factors—social status and level of income. The social status also corresponded with the nature of the working activity—especially in towns, those who did not work manually were considered “better people.” The middle class became the bearer of a new lifestyle in which the emphasis was on health care, education, and reasonably spent leisure time. The middle class consisted mainly of officials employed in the state or municipal administration (their income and the standard of living varied according to their position in the bureaucratic hierarchy) and officials employed in the private sector, such as banking. The middle class also included technical intelligence and businessmen. Since the mid-19th century, the number of doctors, lawyers, and secondary school professors with university education also began to increase. Physical activities were more accessible to boys than girls in large cities. During the 19th century, society's view of the role of women changed, but until the First World War, the majority opinion prevailed that women should realize their potential mainly as housewives and mothers. This dominant masculine attitude made it significantly more difficult for girls to access education and physical activities at school and in associations.

The deficit in physical activities was particularly felt by children and young people living in larger cities. In the period up to the First World War, thanks to industrialization, the cities experienced a continuous development that quickly accelerated at the turn of the 19th and 20th centuries. After the lost Austro-Prussian War, it was decided in Prague to demolish the city walls (the demolition began in the early 1870s), and gradually, many surrounding villages became part of the city. Between 1869 and 1910, the population of Prague grew from 158,000 to 224,000, taking second place after Vienna in the Cisleithanian region. In 1910, 42.5% of Prague citizens worked in industry or trades, 29.7% in public administration, or as soldiers and freelancers, and 27.1% of the population worked in retail and transport. With this modernization of the economy, Prague was approaching the status of the metropolis of Cisleithania, such as Vienna which showed similar sectors of employment (46.6, 25.4, 27.1%) (Jindra and Jakubec, 2015, p. 129–130).

The economic boom in 1867–1873, which attracted many workers to Prague, caused a shortage of flats. Workers lived in colonies, in small houses or, together with tradesmen, rented apartments in tenement houses, which in many parts of Prague formed a monolithic industrial type of construction. The courtyard gallery replaced the 18th-century peristyle with toilets located at the rear end of the gallery. From the gallery, there was direct access to the kitchen, a room of about 24 m2, which served as a kitchen and bedroom for the whole family (Efmertová, 1998, p. 279). The tenement houses were mostly dominated by the spirit of collectivism and mutual help so that children grew up in the collective care of not only mothers but also aunts from the neighborhood. In 1880, women in the Czech lands accounted for 34.4% of all factory workers. Most of them were young single women, those married stayed mostly at home to care for children and because men's wages were on average 40% higher (Jindra and Jakubec, 2015, p. 139).

Dark housing and inadequate hygienic conditions not only brought health risks, but the nature of living and the resulting lifestyle also created a different perception of the outside world, including corporeality, for children from working-class families, as opposed to middle-class children. Members of the middle class—engineers, lawyers, doctors, university, and secondary school professors, senior officials, and small and medium-sized entrepreneurs, lived in apartment buildings of the wider Prague center in multi-room apartments with private bathrooms with hot water.

The state of health of the inhabitants of larger towns, regardless of social stratification and the associated quality of housing, was negatively influenced by the overall poor hygienic conditions of the whole town. Water reservoirs to supply the urban development with utility water were not built in Prague until 1880, and by 1900, most houses were still not supplied with drinking water (Hlavačka, 2016, p. 244).

The standard of living in other industrial centers of the Czech lands differed in its specific forms according to the nature and pace of their industrialization, urbanization plans, demolition of city walls, and the annexation of agricultural communities, which were turned into industrial suburbs. The common problem of all larger cities was the remediation of hygienically completely unsatisfactory parts of the city and the construction of infrastructures, such as water supplies, sewerages, and street-lights (Kota, 2010, p. 16–23). In regards to children's health and their physical development, the living conditions of large industrial centers did not differ much.

## Legal Protection of Children and Youth

The status of children and youth can be seen from two perspectives, first from the perspective of care for children and youth and second from the perceptive of legal capacity. Legal capacity was defined by the “Civil Code of Austria” issued by Emperor Francis I, which came into force in the Czech lands in January 1812 and was effective also in Czechoslovakia until 1950. This legal norm divided people into children (up to 7 years), juveniles (up to 14 years), and minors (up to 24 years) (Tauchen and Schelle, 2012, p. 204). The code did not distinguish people by gender and the age of majority meant getting independence from parental protection. If the father died, the minors were appointed a guardian who had the powers of the father. Protection mainly concerned children under 14 years of age.

The population of the Habsburg Monarchy was young and its future was dependent on the children and youth welfare. That is why the executive power paid so much attention to it, although, whether or not it was sufficient remains to be seen. The sheer vastness of the empire and the differences between countries and regions brought a wide variety of issues. The central government had to share the burden of dealing with these problems with representatives of the provincial and municipal governments, but these governments were given only “adequate powers” which would not jeopardize the integrity of the monarchy. Section 12 of the 1867 Constitution stated that: “All other legislative items not expressly reserved to the Imperial Council in this Act are within the competence of the Provincial Councils of the kingdoms and lands represented in the Imperial Council” (Gesetz vom 21. Dezember 1867, Nr. 141, 1867).

The authority in the area of labor legislation was reserved for central executive power, among others to protect children from child labor abuse in the view of the turbulent pace of industrialization. The Trade Ordinance from December 20, 1859, prohibited the work of children under 10 years and required the employer to care for the minors: “He […] will oversee the morals and behavior of a minor apprentice inside and outside the workshop” and ordered owners of trades and industrial establishments “to refrain from any ill-treatment of the apprentice [and] to protect him against such treatment from other workers.” The Trade Ordinance also required employers “to watch over the school attendance of their wards” (Kaiserliches Patent vom 20. December 1859, Nr. 227, 1859).

The amendment to the Trade Ordinance from 1885 prohibited child labor under 12 years and limited its duration to 8 h per day for children between the age of 12 and 14. Furthermore, it allowed “the use of juvenile workers” aged 12–14 only “if it is not detrimental to health and does not hinder physical development and it does not obstruct the compulsory school obligations” (Gesetz vom 8. März 1885, Nr. 22, 1885). The law was amended again as early as in February 1897. Underage workers could continue to be recruited only based on an employment contract, and because, the apprentices were used only for various domestic jobs, the amendment prescribed to the owners of the trades: “to teach the apprentice the skills of the trade for which he is trained” (Gesetz vom 23. Februar 1897, Nr. 63, 1897). Compliance with these legal norms could not be rigorously monitored. The level of care for minors depended primarily on the goodwill of the employer.

## Morbidity and Diet of Prague Children

From the middle of the 19th century to the First World War, a third of the population of the Czech lands were children up to 14 years of age, young people aged 15–20 represented ~12%, the middle-aged population under 59 years of age declined only slightly (to ~50%), and the group over 60 years grew slightly, amounting to 9% in 1910 (Hlavačka, 2016, p. 207).

The main cause of the low average lifespan was high child mortality, especially new-born mortality. In 1850, half of the children born in Prague did not live to see their first birthday (Hlavačka, 2016, p. 208). In the following years, children died of infectious diseases—smallpox, scarlet fever, typhoid, and diphtheria. Child mortality was the highest among factory workers due to poor hygiene conditions of housing and workplaces.

Despite high child mortality, the 19th century brought a significant increase in population. One of the theories of demographic growth in the 19th century in European countries says that its main cause was an improving diet. It has played its role, but in conjunction with emerging medical and social care, progress in labor law, toward the end of the century also with improvements in urban hygiene, the rising quality of education, and last but not least, awareness of the importance of physical activity for children and youth welfare. In the course of the 19th century, the middle-class diet improved, especially in terms of protein content, including beef, pork, poultry, milk, and eggs. Since the end of the 19th century, however, eating habits had not respected the principles of a healthy diet of today. The people started to prefer bread and pastry made of white wheat flour and the consumption of sugar and animal fats increased. Fruits and vegetables were rather seasonal products (Efmertová, 1998, p. 216). The working-class diet was far from being so varied. Eighty percent of the costs of working-class families were payments for rent, heating, and food. Clothes and other consumer goods were luxuries and the only way to save money for them was by skimping on food. The workers' diet consisted of milk and coffee (it was mostly its substitute made from roasted chicory and later from sugar beet or grain, commonly known as “chicory”), sugar, bread, and porridge or soup made of old bread, potatoes, garlic, onion, and salt. The workers had meat only exceptionally on public holidays, sometimes on Sundays—especially beef, which was first cooked for soup. Dinner consisted of bread with butter or curd cheese or common cheese. Still, roughly ¾ of the workers in the industrial centers did not have enough resources to obtain food in the abovementioned composition or in the amount that would feed their hunger (Efmertová, 1998, p. 269).

The diet given to orphans in orphanages, their nutrition was not much different from their parents. The malnutrition was a significant health risk for a significant percentage of these children. Well-nourished middle-class children, on the other hand, were also susceptible to a dietary danger albeit of a different kind. The physical ideal of the 19th-century child, especially of pre-school age, was a “chubby child,” being slightly overweight suggested the child is healthy, and the absence of a visible fat layer signaled possible health problems. Skinny children were “cured” with small doses of alcohol, such as black beer and ferrous wine (Lenderová and Rýdl, 2006, p. 131).

In 1887, the wife of the then-mayor of Prague Mrs. L. Šolcová convened a “circle of Prague ladies” to address the eating of poor children “who did not have nutritious warm food at noon” (Dolenský, 1920, p. 539). The ladies set up a committee to “provide warm, fortifying food for poor school children” (Dolenský, 1920, p. 546). In 1888 the first canteen was opened. The interest in lunches, however, greatly exceeded the offer, and therefore, the number of canteens increased rapidly and reached the number of eleven in 1897. The municipality had two establishments where food was prepared and then delivered to the canteens. The number of canteens increased with the number of children. There were 300 in the school year 1887/1888, and by the school year 1913/1914, the number reached 1750 (Dolenský, 1920, p. 546). Not all those interested could be satisfied, the eligible children were chosen according to the social situation of the family: “orphans, children of widows and working-class families where children were at lunch […] completely without hot food.” The lunch menu consisted of soup “heavily filled with semolina, noodles, rice or groats, to which each child receives a portion of diced beef. Once a week, the soup is made of peas and once a week a roasted soup is served. With it, the children receive a slice of rye bread or a bread roll each, and on Friday, a very popular cake” (Dolenský, 1920, p. 546).

## Medical Care and Physical Exercise

Child mortality was mainly due to inadequate hygiene conditions at home, at school, in factories, but also hospitals. Children were dying of infectious and other diseases that are difficult to identify because very often, the cause of death was stated as inherited weakness or intestinal catarrh. A major health threat for the entire population in the 19th and partly in the first half of the 20th century was pulmonary tuberculosis. The highest mortality from tuberculosis was recorded in large industrial centers, including Prague, where there was a high concentration of people.

The milestone in the health care of Cisleithania was the adoption of the law “on the organization of public health administration” on April 30, 1870. As was typical for Austrian legislation, the enacted legal norm entrusted the state administration with the “direct authority to keep records of all healthcare personnel and to supervise them in regards to all medical matters …” (Gesetz vom 30. April 1870, Nr. 68, 1870). Nevertheless, the central executive administration was responsible for issuing legal standards “on infectious diseases, endemics, and epidemics [.] on the administration of poisons and medicines and on managing vaccination” (Gesetz vom 30. April 1870, Nr. 68, 1870). The law also entrusted Landtag and municipalities with the supervision of local health care. The stuff was to be proportionate with the size of the region and the number of inhabitants living there. District and municipal doctors with a wide range of responsibilities and powers have become the most important component of the healthcare system.

In 1875, a provincial maternity hospital was opened in Prague, which is still in existence. Outpatient care for pediatric patients began to operate in Prague under provisional conditions in 1888. In 1902, a new modern hospital was opened in Prague. However, only the ground floor with 30 beds for older children was rented to the children's clinic (Havránek, 1998). It follows from the abovementioned facts that the availability of children's hospital care was very low. Private medical practices were also available only to middle-class and upper-class children for financial reasons.

The establishment of an institution of school doctors was essential to the betterment of children's health care. Their task was not to treat children, nor was it possible if we consider their numbers. Their vast contribution to the advancement of the health of pupils and students lay in other duties, such as improvement of school hygiene, mapping of children's health, diagnosing infectious diseases, and then ordering home quarantine to prevent infecting other children at school. The beginnings of medical supervision at Prague primary schools are linked to the decree of the Prague Municipal Council presidium from January 24, 1883, which ordered:

1. Inspection of the cleanliness of the teaching rooms and other premises, including inspection of the heating, ventilation, and cleanliness of toilets
2. Examination of the school children health, including eye examination and control of contagious diseases

The main purpose of the decree was to exempt children who were found to have a health problem (“illness”) from physical education or school attendance, particularly in the case of a rash or “queasiness—inducing illness (discharge from the ear, nose, etc.).” The eyesight inspection and its results, especially myopia, were taken into account when seating children in the classroom (Dolenský, 1920, p. 462).

In 1885, the Mayor of Prague, Tomáš Cerný, submitted a proposal to introduce medical examination of students at primary schools and orphanages, secondary schools, and private schools. Tomáš Cerný, who served as a mayor from 1882 to 1885, was a lawyer and politician with close connections to physical education as he was one of the founding members of Sokol in 1872–1882 and was also its Mayor. In April 1885, the Commission developed two types of programs. The first type was designed for a medical examination at the beginning of school year and included the assessment of growth and body development, eyesight, hearing, hair and skin, spine examination, recording information on past and ongoing illnesses, vaccination, and also an assessment of child's ability to participate in physical education. The second program was for regular check-ups throughout the year and included assessments of school attendance, causes of absence, screening for infectious diseases, assessment of defects in the school building concerning health, and last but not least, physical exercise supervision.

It was stated that: “The children are to be examined carefully outside school hours […] in a special school room in the presence of a teacher, and in the case of a girl, always in the presence of a female teacher, on special request in the presence of parents.” The actual treatment of illnesses was not the responsibility of a school doctor but it was the responsibility of the school administration to inform the parents about their child's illness “in the gentlest way” (Dolenský, 1920, p. 464).

At the beginning of medical supervision in schools in 1883, nine doctors were entrusted with this activity and were assigned 25,531 children at 59 schools (Dolenský, 1920, p. 465). Other milestones in the field of hygiene at schools include the installation of school desks based on student's body height in 1889 and the establishment of showers in 1891 on the proposal of a city physician. In 1880, the office of a city physician was created in Prague. He was a doctor and was responsible for the professional management of municipal health care and also fulfilled the function, in contemporary terminology, of the city hygienist.

The institution of school doctors continued to develop according to several decisions of the City Health Care Commission and the Prague governor from 1901 to 1906. In 1909, there were 12 school doctors and 1 female doctor. Apart from the evaluation of school buildings, their tasks included the medical examination of children (eyesight, hearing, teeth, speech, mental fitness, fitness for physical education, handwork and drawing, etc.) and organization of their vaccination (Dolenský, 1920, p. 465–469).

Almost a fifth of children suffered from malnutrition. In the 4 years (1904–1908), 12.9% of boys and 14.9% of girls in the first grade “suffered from poor nutrition,” and in the second grade, the values grew to 18.7 and 20.3% (Dolenský, 1920, p. 469). In the school year 1907–8, 1,713 boys and 1,678 girls in the first grade were examined. In the same year, 47.3% of boys and 51.3% of girls in the first grade were reported “defective” by school doctors, with the term “defective” probably referring to any health problem (Dolenský, 1920, p. 466).

The recorded results cannot be considered accurate due to the absence of laboratory examinations, but school doctors had considerable experience in the diagnosis of these defects and diseases of children. The health condition of Prague children at the beginning of their schooling was alarming, with practically all indicators showing that girls were a few percentage points worse. We do not have the numbers of students exempt from physical education. However, we can assume that they were not insignificant, which, together with the lack of suitable exercise rooms and only slowly improving levels of hygiene in schools, leads us to the conclusion that the positive impact of physical education on the health of primary school students was low.

Infectious diseases were the biggest health problem for children. According to a general report of school doctors for the school year 1905–6, 67.7% of the children examined in the first grade had an infectious disease. In terms of the frequency of occurrence, these were mostly measles, scarlet fever, chickenpox, whooping cough, diphtheria, and mumps. Early diagnosis and the subsequent isolation of the patient was the only weapon against the spread of the disease. Teachers were trained in recognizing the symptoms and in the appropriate procedures in case they suspected a student to be ill.

The only partially effective way to fight it was to change the environment and stay in the countryside, preferably in the mountains or by the sea. Starting in 1895, children suffering from TBC with a more severe course of the disease were sent to the “Maritime Hospital Ospizio Mario” in Trieste at the expense of the Prague municipality. Between 1895 and 1903, 288 children underwent the treatment which lasted 100 days. Children were examined by a school doctor before and after returning: “The results of the treatment were really surprising. More than half of the children returned healed, others at least improved. These were sent to get the treatment again next year and usually returned healed as well” (Dolenský, 1920, p. 499). From current diagnostics, we can certainly doubt such a high percentage of cured children, but given the experience of school doctors with tuberculosis, we can conclude that a significant improvement indeed occurred.

In the last quarter of the 19th century, efforts were made in the Czech environment to contribute to the improvement of the children and youth health through health exercises. The most notable work in which this effort is reflected is Home Exercise for Girls of Poor Posture by Klemena Hanušová, a pioneer of Czech girls' physical education. The description of the musculoskeletal defects (especially the defective posture) and the exercises to compensate for the harmful consequences of a sedentary lifestyle, which Klemena Hanušová states in 1891, have not lost anything of their relevance today.

The sedentary way of life of urban girls in the second half of the 19th century was not very different from the current one. They had to do housework, such as cooking, cleaning, sewing, crocheting, in which they did not have much movement. At school, as Klemena Hanušová says, the girls were even worse off: “Girls, sitting at school for a long time, start to lounge around from fatigue, especially when writing. They distort their body, and after some time, they lose the sense of straight and correct posture” (Hanušová, 1891, p. 3–4). Klemena Hanušová was a founding member and the first trainer of the “Gymnastics Association of Ladies and Girls of Prague” founded in 1869, where mainly female college students trained. Klemena Hanušová organized and led the training sessions of the association for 27 years.

Adding to the already mentioned fact that the amendment to the School Act of 1883 declared girls' physical education as an optional subject, and therefore, most of them did not participate voluntary, this lack of movement led to muscle weakness (Gesetz vom 2. Mai 1883, Nr. 53, 1883). The author of health physical education observed as early as in 1891 that: “the cause of incorrect posture and incorrect stature is mostly based on muscle weakness. […] Her views correspond to the findings of current physiotherapy.” Today's procedures for treating improper posture, whether preventive or curative, offer a wide range of methods and concepts. However, the active element—physical activity—is evident in all of them. Hanušová understood the meaning of physical activity as related to physical education: “treatment of spinal curvature is done mainly by physical exercise to strengthen muscles and maintain the skeleton in the right direction” (Hanušová, 1891, p. 3–4). In connection with physical education, she considered 1–2 h at school as insufficient and recommended to include at least 3 extra hours of physical activity outside of school. She joined the ranks of enlightened people who realized that the extent and level of school physical education were insufficient for children and young people. Klemena Hanušová recommends regular physical activities in the family environment: “The state of health of young people will not get better unless physical exercise is practiced more often and unless it is practiced in every family and every day” (Hanušová, 1891, p. 6–7). Klemena Hanušová was a founding member and the first trainer of the “Gymnastics Association of Ladies and Girls of Prague” founded in 1869, where mainly female college students trained. Klemena Hanušová organized and led the training sessions of the association for 27 years.

She designed a series of exercises that are diverse and on different levels of difficulty. Some exercises were simple movements of upper and lower limbs, torso or head, others were performed using aids—rod, barbell, box. A large number of exercises were recommended to be performed using a chair, door, bed, or sofa. In these cases, it was recommended to also perform compensating exercises (in today's terminology stretching) to compensate for unilateral overloads or corrected deviations from proper posture. In several exercises, we can see the element of “muscle strengthening.” In some cases, these were quite dangerous—for example, a person lies on two chairs with one supporting only the head and the other supporting half of the calves. All the exercises were divided by difficulty and according to the actual physical condition of the child. There were exercises for “the weakest children,” “adult girls,” and so on. She also recommended the number of repetitions of each exercise as well as a suitable time to perform them: “morning and evening, not immediately before and after meals, in too tight clothes” (Hanušová, 1891, p. 9).

## Physical Education at Primary Schools

Following the December Constitution of 1867, the Austrian Parliament passed many laws to modernize and thus improve the functioning of the Danube monarchy. After the defeat in the Prussian-Austrian War, it was not surprising that the army was not spared these reforms either. The basis for the reconstruction of the army was the universal military conscription: “conscription is universal and every citizen [.], capable of defense, must fulfill it in person” (Gesetz vom 5. Dezember 1868, Nr. 151, 1868). Thanks to the universal conscription duty, in Cisleithania, 470,000 men were to be summoned in the event of mobilization (Gesetz vom 5. Dezember 1868, Nr. 151, 1868). For these soldiers, however, the conscription duty did not end after completing basic military service but they became soldiers in reserve. Obligatory draft, which were strictly controlled, for most conscripts took place after the age of 20, but a significant part of them was exempted from military service for health reason.

Another pillar of the modernization of the Cisleithanian administration was to be the reform of the school system which aimed to transform the whole system of education. Education was to be guaranteed by the state and the state was to be in charge of the decisions regarding the school administration and the framework of the curriculum. The goal was to get the school out of the world of abstract concepts and to prepare pupils and students for life in the real world in the best possible way, especially given their future participation in the labor market. The development of the economy of the Austrian and Czech lands was to become the engine that would stir up the colossus of the Danube monarchy to catch up with the most developed powers, especially Prussia.

In March 1867, the Ministry of Cult and Education was restored. On May 25, 1868, a law was passed “laying down the basic rules on the position of the school to the Church.” Its first paragraph stated the following: “The supreme management of all education and its supervision is the responsibility of the State and will be executed by the authorities designated for that purpose” (Gesetz vom 25. Mai 1868, Nr. 48, 1868). Only the teaching of religion remained within the remit of the Church, and in other subjects, as stated in § 2 of the Act, “the Church [had] no effect.” The Church could set up and administer church schools at their own expense, but church schools had to comply with “issued laws on education,” i.e., they had to comply with all regulations, including the mandatory content of teaching, as state schools (Gesetz vom 25. Mai 1868, Nr. 48, 1868). In short, the letter of the law opened the gate for the creation of a system of modern public education.

The next and fundamental step toward the creation of a new educational system was the adoption of the law establishing the rules for teaching in primary schools in May 1869 (Gesetz vom 14. Mai 1869, Nr. 62, 1869). This law introduced compulsory 8-years schooling for children from the age of 6.

The law stipulated that the primary school “is a public institution” and must be “maintained at the expense of the state, the country or local municipality […] Where and when the obligation to set up a school arises is determined by the provincial legislation,” but it is everywhere “in a one-hour radius where there are more than 40 students (by 5-years average) who have to go to school over half a mile away.” As regards the number of pupils, the letter of the law prescribed that: “if there is an average of 80 pupils in a primary school for 3 consecutive years, a third teacher will inevitably be appointed” (Gesetz vom 14. Mai 1869, Nr. 62, 1869). School councils—provincial, district, and municipal—were established for school management and supervision. The school councils also decided: “Whether pupils are to be separated by gender” (Gesetz vom 14. Mai 1869, Nr. 62, 1869). The creation of co-educated schools was prominent mainly in smaller towns and villages solely for reasons of space and finance. In large cities with a significantly larger number of children, even primary schools were divided into boys' and girls' schools or in one school into classes for boys and girls.

The law furthermore required the public schools to “educate children in morality and religion, develop their spirit, and provide the proficiency they need for further education in their lives” (Gesetz vom 14. Mai 1869, Nr. 62, 1869). The compulsory subjects at primary school included religion, provincial language, calculus, writing, geometry, natural sciences along with geography and history, singing, and also physical exercise. Physical education was supposed to be taught 2 h per week. However, the letter of the law stated: “the extent to which these subjects should be taught will depend on how many teachers will be set up at one school” (Gesetz vom 14. Mai 1869, Nr. 62, 1869). It shows that the extent to which physical education was taught depended on the local conditions and attitude of the school council, which set up teaching posts and selected teachers, which also applied to the school council of Prague municipal council.

According to the law on public schools, 4-years middle schools were also to be established, which “set up to provide further education to those who do not attend secondary school. […] Where and from what funds the middle schools shall be established will be set down by the provincial legislation” (Gesetz vom 14. Mai 1869, Nr. 62, 1869). In Prague in the school year 1875/76, there were 18 boys' and 1 girls' middle school. Workers' children mostly followed in the footsteps of their parents and “were employed in factories or larger trade factories.” For them, “factory owners were obliged to set up schools […] according to the laws on the establishment of public schools” (Gesetz vom 14. Mai 1869, Nr. 62, 1869). Physical education was also among compulsory subjects. However, it was not present at trade and continuing schools.

Compulsory physical education at primary and middle schools was enacted in 1869, but that does not mean that it was taught. In some schools, there was no place to exercise and a qualified physical education teacher, at least at the level of a teacher training institute graduate, was also not always available. However, the material and staffing situation for physical education were continually improving, and in the first decade of the 20th century, boys exercised at the vast majority of schools. In Prague, “for lack of suitable gyms and lack of qualified teachers” (Dolenský, 1920, p. 313), physical education became part of the curriculum in primary schools founded by the city as late as in March 1875. Many meetings and resolutions of the municipal council testify to the fact that: “the Prague municipality assigned considerable importance to the teaching of physical education.” In 1899, Prague councilors decided, among other things, that “when constructing new school buildings, it must be remembered, as far as possible, that the summer gyms would […] be provided with glass walls on at least one side […] so that the walls could be removed in summer and the desired airflow achieved” (Dolenský, 1920, p. 31). Contemporary literature does not mention the extent to which this had been achieved successfully. The glass walls have not been preserved, and even historians of architecture do not know of them. Quite understandably, securing the premises for school physical education took the longest. Few schools had a gym, and in 1911, 21.5% of school districts (Bohemia was divided into 121 districts) did not even have had a playing field (Hanzová, 1992, p. 184).

According to the school regulations for Czech lands from 1870, the goal of physical education was for “young people to gain confidence and courage, to enjoy the orderliness, to have trust in themselves and to maintain the alertness of body and spirit.” The regulation further recommended drills and floor exercises, games, and “at higher levels, where there is sports equipment, also exercises with these” (Hanzová, 1992, p. 41). At primary and middle schools, the teacher had to choose exercises that could be done in modest conditions, mostly in the open air. These were mostly games and gymnastics. The recommended equipment was the one with which it was possible to train in the schoolyard—gymnastic cones, skipping ropes, short sticks, or which could be installed in the absence of the gym, such as ladders. The physical education of boys and girls was similar, while the girls did not engage in strength training, such as climbing, which was recommended only for boys. From the end of the 19th century, teachers in primary and middle schools also had an adequate number of manuals for teaching physical education containing a selection and description of exercises suitable for the physical education of children at public schools.

## Preparing Teachers for Primary Education

The imperial law on primary schools also enriched the education system of Cisleithania with a unified system of education of teachers who were supposed to work in primary and middle schools. The executive power retained the control over the education of teachers who were to educate children and youth in the future as the organizational framework and content of teacher training institutes remained fully in the hands of the Minister of Cult and Education. The representatives of the Austrian government seemed to realize that the contemporary form of child upbringing within the families was strongly influenced by belonging to the social strata, and because of the growing nationalism, the ethos of respect for the reigning family disappeared and was replaced by upbringing which led to the creation of purely national identity. The school thus remained the last and key institution that led or was supposed to lead the children to feel solidarity for the Habsburg House.

A girl or boy who “reached 15th year of his age, was of a healthy and brave body, of integrity, and had a proper preparatory education” could become a participant in a 4-years training course, “separated by gender of partakers.” He or she had to prove this in the “rigorous examination of subjects taught at lower secondary schools or lower gymnasiums except for foreign languages” (Gesetz vom 14. Mai 1869, Nr. 62, 1869). The curriculum of compulsory subjects at boys' teacher training institutes included religion, pedagogy, mathematics, introduction to natural sciences of physics and chemistry, geography and history, citizenship education, farm economics, writing, drawing, music, and last but not least physical education (Gesetz vom 14. Mai 1869, Nr. 62, 1869). The composition of compulsory subjects at girls' institutions was similar, including physical education. Instead of the foundations of farm economics, the girls were taught “home economics” and “handwork,” and unlike boys, also the languages that “the Minister of Education determined on the proposal of the Provincial Office.” At the end of the fourth year, the students had to pass a “rigorous examination” of all subjects taught and if he/she was successful he/she received a “maturity certificate,” i.e., graduated. In boys' institutions, a primary school had to be set up for students' practice, and girls' teaching schools were linked to a children's garden (Gesetz vom 14. Mai 1869, Nr. 62, 1869).

The special attention and care given by the Austrian government to teacher education are illustrated by three factors:

1. Studying at teacher training institutes was free of charge and “poor, mentally gifted inmates (i.e., students) receive scholarships when they undertake to serve as a teacher for at least 6 years.”
2. There were not to be more than 40 students in one class of teacher training institutes.
3. The law on primary schools introduced a system of lifelong learning for teachers, which included compulsory attendance at professional conferences and the offer of post-graduate courses “at the time of the autumn holidays” (Gesetz vom 14. Mai 1869, Nr. 62, 1869).

In the second half of the 19th century, the exclusive conditions and high demands placed on students at teacher training institutes shifted these educational institutions into the field of exclusive education on the same level as gymnasiums. No tuition and the possibility of receiving scholarships opened the gates of these schools to talented, hardworking, and motivated students from lower social classes.

On May 26, 1874, the Ministry of Cult and Education issued an organizational statute for teacher training institutes. In this comprehensive regulation, physical education was given considerable attention. For boys' teacher training institutes, the objectives of the exercise were formulated rather ambitiously: “To perform dexterity in an exemplary manner, […] to recognize the mechanisms of movements and changes in their development, to understand the technique of movements, to acquire the ability to decompose them, […] to become acquainted with the historical development, with the principles and with the pedagogical task of physical education” (Hanzová, 1992, p. 132).

Furthermore, the aforementioned organizational statute defined the content of physical education in individual years, especially didactic procedures, and stated that only in the third year should the “grater consideration be given to individual exercises to increase students' dexterity” (Hanzová, 1992). Within the female teacher training institutes, the organizational status defined the same goals for physical education, but how they were to be achieved was somewhat different. Greater emphasis was put on group exercises and practical and theoretical instructions focused on physical education for girls.

A student could be excused from physical exercises only based on a medical certificate, but he/she was not exempt from watching physical education lessons and theoretical physical education lessons. The curriculum of physical education seemed to be well-thought-out and modern. However, the number of lessons per week, i.e., 2 h of physical education per week in the first 2 years, and a lesson per week in the third and fourth years, which were lesser than the violin lessons, were probably not enough to achieve the skills of future primary school teachers.

On May 2, 1883, a law was adopted “amending certain provisions of the law of 14 May 1869.” It brought one important change in the teaching of physical education at primary and middle schools and that was the fact that physical education remained compulsory only for boys, for girls it became “non-obligatory” (Gesetz vom 2. Mai 1883, Nr. 53, 1883). The amendment also allowed an increase in the number of pupils in the class to one hundred during half-day classes. The amendment to the Primary Schools Act gave the school councils room to specify some of its sections. The municipal school council in Prague decided, “that voluntary physical education at girls' schools should always be put at the end of lessons or in the afternoon” (Dolenský, 1920, p. 276).

The annual report of the Provincial School Council for Bohemia for the school year 1910/1911 confirms that physical education for girls was taught at primary and middle schools only exceptionally because of “lack of suitable premises or [because] the parents of pupils did not want it to be taught.” The negative attitude of parents toward the physical education of girls led to the fact that “not all pupils participated in the teaching of this optional subject […] nor even at schools when there is the opportunity to do so” (Hanzová, 1992, p. 184). The parents' negative attitude to non-compulsory physical education could have been caused by their inclusion at the end of their classes, as mothers might have preferred to have their daughters rush home after school and do housework instead of physical education.

## The Position of Physical Education in Secondary Education

Secondary schools underwent evolutionary development from the mid-19th century until the First World War. There were several causes of the relative conservatism of secondary education, but the elitist nature of this level of education dominated among them. The Czech ethnic environment perceived the growth in the number of secondary schools with the Czech language of instruction as a success of national emancipation efforts. Furthermore, the establishment of the gymnasium strengthened the prestige and importance of the town where it was founded. Graduating from secondary school opened up opportunities for employment outside of manual work, which in itself brought a higher social status.

But the desired social status was not free. Secondary school students had to acquire a considerable amount of knowledge and submit to a discipline that consisted of meticulous homework and orderly behavior in the classroom.

The framework of secondary education in Austria and Austria-Hungary was shaped by Proposal on the Organization of Gymnasiums and Secondary Schools in Austria (Orig. Entwurf der Organization der Gymnasien und Realschulen in Oesterreich), written by University professor of philosophy and the Austrian ministerial councillor Franz Serafin Exner and the German gymnasium professor and philologist Hermann Bonita (Rezníčková, 2006, p. 13). The principles of the proposal began to be applied in 1854 and created the pillars on which Austrian secondary education had been based throughout its upcoming existence. The reform significantly reduced the influence of the Church and strengthened the role of state and provincial authorities in the establishment of secondary schools and administration and the authority of the director and the teaching staff in their day-to-day management. Gymnasiums had 8 years of instructions divided into two stages—lower and higher, the lower gymnasium could exist separately (Rezníčková, 2006, p. 14). The content of the curriculum was supposed to help bring the graduates closer to real life by extended teaching of science subjects. However, the study of Latin and Greek still accounted for almost half of the curriculum of compulsory education. The state graduation exam was introduced and successful completion of this very rigorous exam became a prerequisite for admission to the university or the state administration.

Another type of secondary school was a “Realschule” which was similar to the gymnasium in the number of years of instruction. Graduates of Realschule could study at the Technical University in Prague or Vienna. Learning at these schools was mainly supposed to prepare students for employment in various jobs in public administration and the private sector after graduation. In 1868, the length of Realschule studies was extended to 7 years, and a year later students took their first school-leaving exam. The December Constitution of 1867 did not include their administration in the imperial competences, and thus their establishment and management were transferred to the competencies of the territorial assembly, or rather the provincial school councils. Physical education was mostly taught at Realschule because the provincial school council could classify it as a compulsory subject.

When designing the Proposal on the Organization of Gymnasiums and Secondary Schools in Austria, its creators considered the inclusion of physical education as a compulsory subject. In the end, it was incorporated into a group of elective subjects, which students had to pay for beyond the tuition fee. For that reason, there was no uniform curriculum for school physical education, and each institute, if it wanted to teach physical education, created it separately and submitted it to the provincial school council for approval (Rezníčková, 2006, p. 82).

Most Catholic priests opposed physical education in secondary schools, and a large number of secondary school professors believed that a student of the Austrian gymnasium should study hard and not run around the playfield. However, some gymnasiums taught physical education before the introduction of the Exner-Bonitz reform. At gymnasiums where physical education was taught, the schedule was mostly in the afternoon after completion of compulsory subjects. The gym could be found in school buildings only exceptionally, and therefore students usually spent the lesson in the open air. They practiced floor exercises, and accompanied by the teacher, made trips to the surroundings of their school. Physical education lessons thus largely depended on the weather and local conditions—in winter, physical education was taught only at schools where the surroundings offered opportunities for skating and sledding.

Vocational secondary schools started to emerge in the middle of the 19th century. There were industrial, commercial, and economic (agricultural) schools, where physical education was mostly not part of the curriculum.

At the turn of the 20th century, some secondary school teachers began to point out the miserable physical condition of the students caused by the lack of exercise and fresh air. Professor Karel Kopecký, a teacher at a gymnasium in Rychnov nad KněŽnou, wrote in 1904: “In the youth entrusted in their care, the teachers observe pale cheeks, stunted growth, humped posture, frequent headaches, sore throats, nose bleeds, the weakening of their mental power and energy for learning” (Rezníčková, 2006, p. 84). The poor physical condition of gymnasium students meant higher sickness rate and lower work performance of future officials and negative social and economic impacts on the activities of middle-class members in education, health care, justice, and private entrepreneurship. Poor health and physical condition of secondary school students also often led to an exemption from military service. Although graduates of secondary schools formed only a small group of the population, they were rather significant in terms of potential mobilization of military reserves. According to the aforementioned conscription law (§ 21), after graduation from secondary school or university, the students could volunteer for a 1-year military service, which was advantageous because the other conscripted soldiers served in the army for 3 years. These 1-year volunteers passed the officer's test at the end of their service in the army and after leaving active service became officers in reserve (Gesetz vom 5. Dezember 1868, Nr. 151, 1868). In the event of mobilization, they would be called to supplement the professional officer corps as necessary. Quite understandably, a secondary school graduate who was “not fit in terms of spirit and body” could not fulfill the conscription duty (Gesetz vom 5. Dezember 1868, Nr. 151, 1868, p. 400). Although probably “fit in the spirit” as the owner of the school leaving certificate, the recruitment committee quite often assessed his physical condition for service in the army as unsatisfactory.

The Austrian Ministry of Cult and Education invited education experts to carry out the reforms and in 1908 organized a survey on the reform of secondary school. Based on the results of this survey, physical education became a compulsory subject in gymnasiums in 1909 in the amount of 2 h per week.

The content of teaching at gymnasiums fell within the competence of the imperial Ministry of Cult and Education, and therefore the curriculum, which was designed for 8-years-long gymnasiums, came into force by ministerial decree in June 1911. The goals of physical education were declared as follows: “Versatile and uniform body development. Maintenance and strengthening of health. Acquiring a natural, beautiful posture. Education toward the conscious will-controlled movement. Body strength and dexterity. Sensitivity, mental alertness, and joy. Courage, prudence, perseverance. Sense of order and sociability. Stimulating a lasting taste for body strengthening” (Učebná Osnova, 1911).

The teaching of physical education according to the new curriculum was based on the principle of gradual addition of different physical activities and an increase in their intensity. Essentially, this meant that demands on students in terms of physical and motor skills were gradually increasing. Students of gymnasiums were supposed to learn floor and drill exercises, as well as exercises on apparatus, play games, and practice so-called “folk exercises,” which included walking, running, jumping, throwing and catching, batting, and tugging. In the fourth grade, basic fencing exercises (advance, retreat, lunge) were added, as well as exercises with light dumbbell and iron bars. From the sixth grade, the dumbbells were little heavier (2 kg) with which the students circled and lunged. They also practiced with a two-pound rod as with a self-defense tool (Učebná Osnova, 1911).

On September 10, 1870, the Minister of Cult and Education established an examination committee in Vienna for those who wanted to “become a teacher of physical education at a secondary school.” In the theoretical exam, the candidate for physical education teacher at a secondary school had to prove knowledge of the history of physical education, knowledge of the works of “Jahn, Eiselen, and Spiess” as well as knowledge of exercise apparatus and “how to establish exercise stations.” Another part of the theoretical examination focused on the candidate's knowledge of anatomy and physiology and first aid. During the practical exam, the candidate had to demonstrate his skills in “floor and apparatus exercises” (Verornung des Ministers für Kultus und Unterricht vom 10. September 1870, Nr. 116, 1870). In 1879, the same examination committee began to operate in Prague.

In 1871, a 2-years training course was set up in Vienna for future physical education teachers. In 1892, a similar course was open in Prague. The courses were held for four semesters and their content corresponded to the topics of the above-mentioned exam. In 1906, the Czech course was connected to the Institute of Anatomy of the Faculty of Medicine of Charles University, and the position of the course director was assumed by the university professor, usually a professor of anatomy. This “promotion” of the course brought an increase in its prestige and led to a better quality of teaching. However, if a graduate wanted to work as a full professor at secondary school, he had to study another subject at least eight semesters at the Faculty of Arts of Charles University.

## Conclusion

This article focused on the development of health care and the physical growth of youth in Prague, the metropolis of the Czech lands, in the period after the division of the Habsburg monarchy into the Cisleithanian and Transleithanian regions. The fundamental legislative framework for this agenda was gradually created through laws enacted by the Imperial Council, which were valid throughout the territory West of the Leitha River. This legislation included labor law, laws on education, and health care. The concept of the modern constitution of 1867, which guaranteed the civil rights and freedom of the individual, was based on the division of power between the central executive and legislative power and the provincial and municipal bodies. Especially in education and health care, a considerable part of the competencies was entrusted to the territorial assemblies and municipalities, which was the right decision in such a large and unevenly developed area, such as Cisleithania. Vienna was motivated by an effort to modernize the entire Cisleithanian region, which was extremely difficult. Eighteen countries formed a very disparate unit with industrially developed areas—such as Lower and Upper Austria and Bohemia, Moravia, and Silesia, alongside very underdeveloped regions—such as Galicia and Bukovina. Although Prague did not reach the economic significance and cultural level of Vienna, which was the center of the entire Cisleithanian region, it was the most developed provincial metropolis. Especially in the period studied, Prague became a regional center marked by the rapid development of industrialization with corresponding rapid urbanization. This negatively affected the environment in the city, which harmed the health of children and youth and limited the possibilities of their physical activities. We focused on school physical education, which was the only opportunity for regular physical activity for most of the children and young people in Prague until the end of the 19th century. School physical education, as well as the work of school doctors, had its limits, but the Austrian, or rather the Prague school showed one of the key paths for the physical development of children and youth. On the example of Prague, we can see that Prague councilors paid considerable attention to the welfare of children and young people but it never became a part of the political struggle. From the 1860s, the Czechs controlled the Prague City Hall, and all councilors agreed to support Czech education. The rivalry between the Czechs and the Germans was an advantage for the welfare of children and young people because the social elites of both nationalities paid considerable attention to it. Also, at the provincial and imperial levels, the children and youth welfare was not a very politicized topic, and most representatives of the legislative and executive powers were guided by the effort to develop it with rational tools. The well-intentioned effort came up, quite understandably, against the material condition. A vast majority of the population of the relatively “rich” Cisleithanian region lived in modest to poor conditions, and only the absolute elite of society was truly affluent. Public budgets also had limited resources. This also applies to the Bohemian territorial assembly and the Prague municipality as both were responsible for the material part of children and youth welfare.

At the end of the 19th century, there was already a decent awareness within society about the necessity of physical activity for children's health and physical development and the importance of physical activities in the lives of young people. In most cases, however, more important and necessary areas worthy of support emerged in the care of children and young people. It was, and probably still is, true that: “everyone knows the refreshing effect of physical education, especially games; nobody doubts that properly conducted exercise prevents the emergence of various school diseases and yet exercise is still neglected!” (Bešták, 1895).

Between the mid-19th century and the outbreak of the First World War, the Cisleithanian part of the Habsburg monarchy underwent a process we call the transformation of traditional society into modern. The goal of our study was to analyse the conditions in which children and young people grew up—mainly in terms of health: where they lived, what they ate, what education and health care they received, and what physical activities they could do. The period we have studied starts in 1867 when a new constitution was adopted by the Austrian Parliament. This constitution divided the monarchy into two units in terms of state administration and made Cisleithania a modern decentralized parliamentary state. In 1869, the Law on Primary Schools brought a revolution in the education of the entire population, and what is crucial for our study, made physical education a compulsory subject within primary education. Conducting our research for the whole area of the western part of the Danube monarchy would require much more space than an academic journal allows us, and therefore, we have concentrated on Prague, as an example of a dynamically developing industrial center.

## Data Availability Statement

The raw data supporting the conclusions of this article will be made available by the authors, without undue reservation.

## Author Contributions

MW and DP took part in the writing of this paper. Both authors have made a substantial, direct and intellectual contribution to the work and approved it for publication.

## Conflict of Interest

The authors declare that the research was conducted in the absence of any commercial or financial relationships that could be construed as a potential conflict of interest.
